# Conservative Medical Management of a Tracheal Perforation after Blunt Trauma in a Patient with SARS-CoV-2

**DOI:** 10.1155/2022/7344476

**Published:** 2022-05-02

**Authors:** Mateo Zuluaga-Gómez, Daniel González-Arroyave, Carlos M. Ardila

**Affiliations:** ^1^Hospital San Vicente Fundación, Rionegro, Colombia; ^2^Universidad Bolivariana, Medellín, Colombia; ^3^Universidad de Antioquia, Medellín, Colombia

## Abstract

This article reports the case of a woman with tracheal perforation due to closed neck trauma and the presence of SARS-CoV-2. The physical examination revealed subcutaneous emphysema in zone II of the neck. The tomography revealed an anterior and proximal tracheal lesion, a 2-mm solution of continuity of the anterior infraglottic airway in the proximal third with subcutaneous emphysema and a decrease in the diameter of the airway at the level of the glottis. The PCR result for SARS-CoV-2 was positive. The medical procedure consisted of orotracheal intubation to guarantee the safety of the airway, in addition to close surveillance in the intensive care unit and constant monitoring of vital signs. In tracheal perforation due to closed neck trauma, it is recommended to evaluate the clinical parameters periodically, including the stability of respiration and subcutaneous emphysema.

## 1. Introduction

Tracheobronchial injuries occurring after penetrating or blunt trauma are rare, although unfortunately they can be life-threatening. It has been indicated that a high percentage of these patients die before being admitted to the hospital, and a significant number die within the first hours after admission [1]. Penetrating injury to the airways can occur from sharp objects, or from wounds firearm injury [2], while iatrogenic airway injury can occur during surgery, endotracheal intubation, or bronchoscopy [3]. However, the incidence of airway injury is likely to be underestimated since these injuries are often poorly recognized and reported [4]. According to some case reports, the therapy of choice for these tracheal injuries is early surgical repair. However, conservative management using endotracheal tubes has also been reported [4, 5]. This article reports the case of a patient with tracheal perforation due to closed neck trauma, and the presence of SARS-CoV-2, who had conservative medical management through orotracheal intubation.

## 2. Case Report

The case of a 42-year-old female patient with no medical history is described. She was admitted to a high-complexity hospital after suffering a traffic accident. The patient presented amnesia of the event and an altered state of consciousness. She also presented facial trauma, closed trauma to the neck and left arm, and lacerations to the lower extremities.

**Figure 1:**
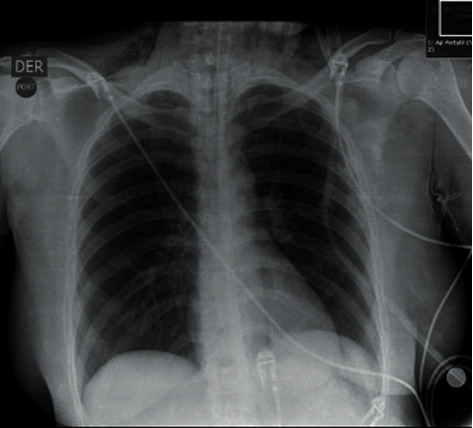
Chest X-ray: emphysema is observed in the cervical region, the cardiothoracic index was within normal limits, and no rib fractures, pneumothorax, or pleural effusion are identified, without consolidations or interstitial infiltrate.

Upon her admission, the following vital signs were observed: blood pressure 130/64 mmHg, heart rate 90 per minute, respiratory rate 17 per minute, and oxygen saturation 98%. The physical examination revealed an alert patient, oriented in the three spheres, with left periorbital ecchymosis, wound in the upper eyelid on its medial side, with little active bleeding and limited eye-opening. She was immobilized with a rigid collar, with a nonpulsatile hematoma in the neck area, with pain on palpation in the left lateral face, and with subcutaneous emphysema in zone II of the neck. Moreover, she had no stigmata of the chest (Figure 1) or abdominal trauma.

High-risk mild cranial brain trauma was suspected with a left orbital fracture, without compromise of visual acuity, with closed cervical trauma, with soft signs of the airway and/or vascular injury. Initially, continuous monitoring was requested, intravenous fluids (500 cc bolus), angiotomography of neck vessels, and tomography of the paranasal sinuses and face, in addition to contrast-enhanced tomography of the chest and abdomen. Administration of tetanus toxoid, analgesics, and paraclinical were also ordered. Furthermore, the patient incidentally reported 5 days of dry cough, no runny nose, no fever, no signs of respiratory distress, and no diarrhea. However, due to the SARS-CoV-2 pandemic, an antigen test and polymerase chain reaction (PCR) were requested to detect the virus. The patient was isolated in the acute respiratory care unit.

**Figure 2:**
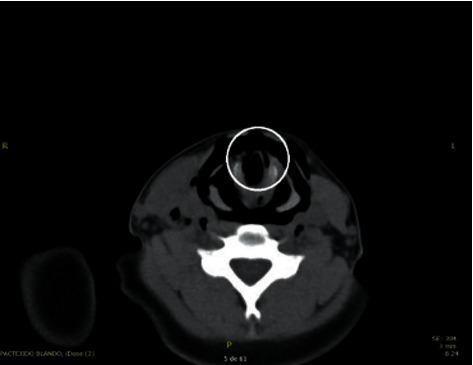
A 2 mm continuity solution of the anterior infraglottic airway is observed.

**Figure 3:**
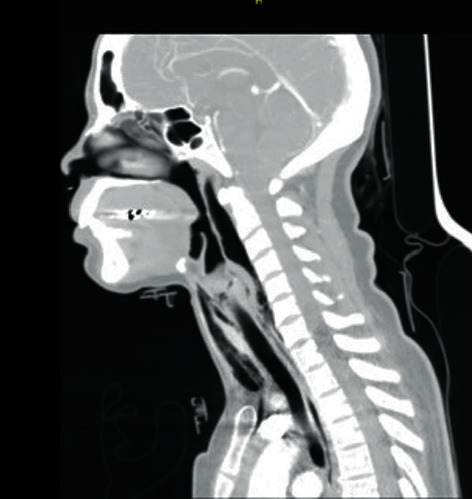
Subcutaneous emphysema is observed dissecting the visceral, carotid, and prevertebral space and causing anterior and middle pneumomediastinum, with a decrease in the diameter of the airway at the level of the glottis.

**Figure 4:**
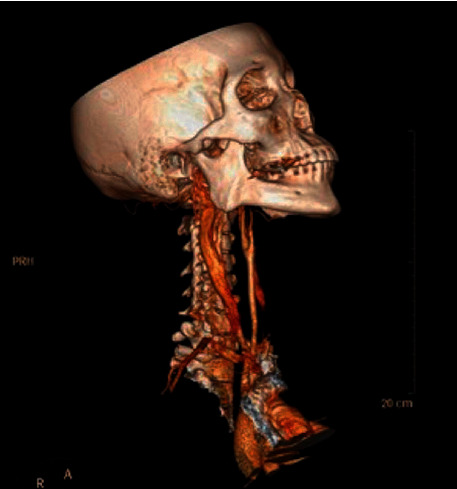
No lesions are evident in large vessels of the neck.

The computerized axial tomography (CT) of the neck, thorax, and abdomen showed anterior and proximal tracheal injury and dissolution of continuity of 2 mm of the anterior infraglottic airway in the proximal third (Figure 2), with emphysema subcutaneous dissecting the visceral, carotid, and prevertebral space and causing anterior and middle pneumomediastinum, with a decrease in the diameter of the airway at the level of the glottis (Figures 1–3). The tomography of the paranasal sinuses showed fractures of the nasal bones, local and left periorbital edema. Simple skull CT showed no pathological findings. No lesions were found in large vessels of the neck (Figure 4). It was decided to intubate the patient on an emergency basis in the hands of anesthesiology with all protective measures in the context of a patient suspected of SARS-CoV-2 infection.

The patient was hospitalized in the intensive care unit due to anterior and proximal tracheal injury of the anterior infraglottic airway in medical management with orotracheal intubation. In addition, the result of the PCR for SARS-CoV-2 was positive, an issue that could complicate the outcome of the case. The medical conduct consisted of orotracheal intubation to guarantee the safety of the airway, in addition to close surveillance in the intensive care unit and constant monitoring of vital signs. During clinical surveillance, there was no evidence of an increase in subcutaneous emphysema, nor the presence of signs of respiratory distress.

In these cases, bronchoscopy should also be performed to confirm the diagnosis and see the injury site. However, considering that the patient was infected with SARS-CoV-2, the bronchoscopy was not performed because the risk of contagion could be increased during the procedure. Moreover, at that time, the patient already showed signs of improvement.

The patient was discharged on the eighth day after her admission with anterior and proximal tracheal injury and nasal fractures; she was given follow-up and outpatient rehabilitation.

## 3. Discussion

In the present case, the conservative medical management of a tracheal perforation after blunt trauma in a patient diagnosed with SARS-CoV-2 is described. Although the tracheobronchial injury is more common among women, men tend to have a higher risk of mortality [6]. Moreover, the most frequent cause of tracheobronchial injuries due to blunt trauma is motor vehicle accidents [7].

As observed in the present case, the typical findings in the context of tracheobronchial injury include subcutaneous emphysema, pneumomediastinum, and pneumothorax [8]. In the diagnosis of tracheal injury, the degree of clinical suspicion, CT, and bronchoscopy are helpful. Radiographic images may reveal pneumomediastinum, subcutaneous emphysema, pneumothorax, or the tracheal tear itself [9].

The treatment of tracheobronchial injury in most cases must be individualized, depending on the patient's comorbidities, the clinical presentation, and the anatomy of the tracheobronchial injury. As in the present case, it has been described that in tracheobronchial lesions involving the mucosal layer with mediastinal or subcutaneous emphysema (level II lesions), patients are treated according to their manifestations with nonsurgical therapy. However, it has been reported that if managed conservatively, tracheal perforations are associated with a 10-fold increase in mortality [7], although in the absence of progressive deterioration, open lesions, or mediastinitis, conservative treatment has not been associated with increased mortality [10].

As described in this report, it is recommended to periodically assess clinical parameters, including stability of respiration and subcutaneous emphysema [4].

The prognosis for the patient with tracheobronchial injury depends on several factors related to the underlying clinical status of the patient, the degree of the tracheobronchial injury, and the type of repair [4]; consequently, the prognosis of the patient in the present case was good. However, it is necessary to highlight that when a tracheal perforation occurs, in addition to an infection by the SARS-CoV-2 virus, there could be a greater risk of causing a ventilatory failure that could cause complications such as severe pneumonia due to COVID, a longer stay in intensive care unit and even death.

## Data Availability

The clinical data utilized in this report are described in this article.
